# Studies of Simultaneous Friction and Corrosive Processes in the Presence of Abrasive Particles

**DOI:** 10.3390/ma15196734

**Published:** 2022-09-28

**Authors:** Przemyslaw Tyczewski, Karol Nadolny, Wieslaw Zwierzycki, Dariusz Ulbrich

**Affiliations:** Faculty of Civil and Transport Engineering, Institute of Machines and Motor Vehicles, Poznan University of Technology, 60-965 Poznan, Poland

**Keywords:** friction, wear, corrosion, abrasive wear, synergy

## Abstract

Providing high-quality machinery and equipment in technical terms is an activity aimed primarily at ensuring the high reliability of nodes. Reliability of machinery and equipment is mainly characterized by resistance to destructive processes. Mutual displacement of parts causes destructive friction phenomena, the intensity of which can be intensified by the interaction of specific technological environments. The article presents the results of research on ring-on-ring samples made of C45 steel in the non-heat-treated state, which were subjected to mechanical, corrosive, and abrasive wear and various combinations of them. The main purpose of the study was to determine the wear that results from the action of destructive friction and corrosive processes with the presence of abrasive material in the tribological node. The results supplement the knowledge of material wear under the simultaneous action of several destructive factors. Based on the study, it was noticed that the sum component of mechanical–abrasive–corrosive interactions is about 40–50% of the total wear. Mass loss resulting from simultaneous mechanical and abrasive interactions is equal about one-third of the total wear. In addition, it was observed that the effects of the interactions of friction (mechanical), corrosive and abrasive excitations are synergistic in nature, which lead to increases in the total wear of the tested samples made of steel. The results of the research are of practical importance and allow for wear-optimal selection of material in the friction node of modern machinery and equipment.

## 1. Introduction

Machinery and equipment manufacturers are striving to reduce the toxic compounds produced by these machines [1,2], as well as to extend their trouble-free life in order to avoid breakdowns and additional repair costs [3]. All these actions lead to protecting the natural environment. The variety of technological environments found in food processing machinery and equipment means that components of moving nodes are subject to extremely rapid wear under the simultaneous effects of friction, thermal, and corrosive forcing [4,5,6,7,8,9]. Due to the variability in operating conditions, the wear of equipment components varies. For this reason, it is very important to have an in-depth understanding of the destructive processes and phenomena occurring in the nodes of these machines. Their negative effects can be more effectively counteracted, as well as consciously controlled—designing the lifetime of the machine.

Research on the wear process on machine components has been carried out for many years [10,11]. Researchers primarily seek to reduce the effects of wear, which can be caused by friction [12], corrosion [13,14], or the impact of additional abrasive particles [15]. In practice, it is rare that there is only one determinant of wear. Usually, there are several of them, which can lead, for example, to corrosion–mechanical wear of mutual machine parts. This is a situation in which the process of mechanic wear is accompanied by the corrosive effect of the external or technological environment. A. Stachowiak [16,17] presents the results of tribocorrosion studies of materials that do not exhibit passivation. In this article, a selection of components of the wear process were separated into: a purely mechanical component, a purely corrosive component (corrosion without friction), and the synergistic effect of friction and corrosion. The article [18] illustrates the influence of material properties (hardness, corrosion resistance) on the intensity of tribocorrosion in the form of wear maps to facilitate the selection of a material for a specific application in a node. Madsen [19] conducted experimental studies with only abrasive or corrosive processes and tests with simultaneous occurrence of abrasive and corrosive processes. These studies mainly dealt with the interaction between abrasive and corrosive wear processes, and the wear value was expressed as material loss per unit time in mm^3^/h. Similar studies were carried out by Nesic et al. [20], in which the synergistic effect of erosive and corrosive action on steel was determined. Zheng et al. [21] dealt with erosion–corrosion processes by studying stainless steel in a corrosive environment. The authors concluded that the erosion and corrosion processes probably intensify each other, causing greater material loss than under conditions when the processes occur separately. Another example of testing the synergistic effect of wear are tests under dry friction conditions and under liquid friction conditions in distilled water [22]. The test specimens were made of Fe_3_Al alloy, and material loss under mixed friction was less than under dry friction at the same load.

Krbata et al. [23] showed that an increase in friction velocity significantly affects the damage and degradation of the surface of the test specimen made of tool steel. There is also an area of research that lead to modifying the surface layer to achieve certain properties, including reducing wear [24,25]. The authors of article [26] used atmospheric plasma spray process to produce layers that have good adhesion to C45 steel and improve hardness in relation to base material. Szala et al. [27] compared abrasive wear resistance of C45 to other steel. It was concluded that C45 carbon steel was less resistant than AISI 304 for r garnet, corundum, and carborundum abrasive material. Tests of the corrosive wear behavior of stainless steel in sliding condition were also conducted [28], and the authors conclude that the increase in the corrosion ratio accelerates the wear of the steel.

Despite the fact that in the literature there are results available from studies of wear of various materials, especially for situations involving the interaction of several types of wear, the problem of determining the individual types of wear (abrasive, mechanical, corrosive) in total wear has not been fully solved. In technical reality, especially in many nodes of machines of the food industry, there are effects resulting from the simultaneous influence of destructive mechanical, abrasive, and corrosive factors, often leading to their failure. Rational action to reduce these effects requires knowledge of the mechanisms of these processes. The phenomena are not complex only because of the simultaneous occurrence in the contact area of several fundamental processes of a different nature (tribological, corrosive) but also because of the possibility of their mutual interaction.

The purpose of the research presented in the article is to determine the quantitative relations between (wear) effects, which are the effects of simultaneous action of destructive friction and corrosive processes, with the occurrence of interactions of the solid phase acting as an abrasive in tribological nodes. The research presented in the article allows to determine the final wear, which can—depending on the operating conditions of the node—be higher or lower than the sum of the individual components of material wear. This will allow us to know the quantitative relations between the effects of simultaneously occurring destructive processes and thus enable better design of similar nodes in the future.

## 2. Materials and Methods

**Figure 1:**
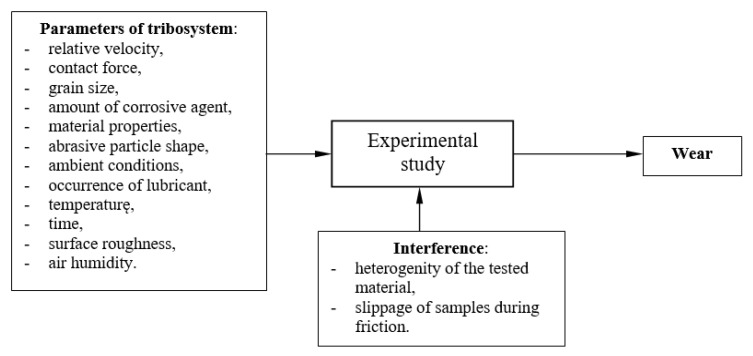
Scheme of experimental research.

After a detailed analysis of the issue of wear of friction node and an assessment of the relevance of the input function (mechanical, abrasive, and corrosive), an experimental study was planned taking into account the input factors, constants, and interferences that may occur during the implementation of the study (Figure 1). The study included four input factors, which varied at four levels as follows:

x_1_—relative velocity rpm, 0 < x1 < 140;
x_2_—contact force N, 0 < x2 < 40;
x_3_—grain size mm, 0 < x3 < 0.3;
x_4_—amount of corrosive age nt %, 0 < x4 < 8.

To carry out the experiment, 125 measurements were made, 25 tests with five repetitions for each of the assumed variants was conducted.

The theoretical model describing wear (I_T_) can be presented as the sum of components resulting from mechanical (I_M_), corrosive (I_C_), abrasive (I_A_) actions, and the effects of their interactions (I_Δ_):

I_T_ = I_M_ + I_C_ + I_A_ + I_Δ_ (1)

The interaction component I_Δ_ needs to be explained in detail. This component can be presented as the sum of components resulting from such destructive processes as abrasive–mechanical (I_ΔAM_), corrosive–mechanical (I_ΔCM_), abrasive–corrosive (I_ΔAC_), and the simultaneous influence of abrasive–corrosive–mechanical processes (I_ΔACM_):

I_Δ_ = I_Δ__AM_ + I_Δ__CM_ + I_Δ__AC_ + I_Δ__ACM_ (2)

The abrasive–mechanical interaction depends on the effect of the abrasive agent on mechanical processes (I′_ΔAM_) and the effect of the mechanical agent on abrasive processes (I′_ΔMA_):

I_ΔAM_ = I′_ΔAM_ + I′_ΔMA_ (3)

Similarly, the corrosion–mechanical interaction depends on the effect of the corrosive agent on mechanical processes (I′_ΔCM_) and the effect of the mechanical agent on corrosive processes (I’_ΔMC_):

I_Δ__CM_ = I′_Δ__CM_ + I′_Δ__MC_ (4)

In addition, the abrasive–corrosive interaction depends on the effect of the abrasive agent on corrosion processes (I′_ΔAC_) and the effect of the corrosive agent on abrasive processes (I′_ΔCA_):

I_Δ__AC_ = I′_Δ__AC_ + I′_Δ__CA_ (5)

By substituting the formulated relations into Equation (1), obtains a model in the most generalized form:

I_T_ = I_M_ + I_C_ + I_A_ + I′_Δ__AM_ + I′_Δ__MA_ + I′_Δ__CM_ + I′_Δ__MC_ + I′_Δ__AC_ + I′_Δ__CA_ + I_Δ__ACM_ (6)

All the results of wear due to various destructive factors included in equations 1–6 are given in g. The determination of the mechanical (I_M_) and corrosion (I_C_) components can be made by performing experimental tests that ensure the occurrence of only mechanical or only chemical input factor. The occurrence of abrasive wear requires the abrasive to be brought to the cooperating surfaces. Contact of the abrasive with the surface (during friction) can take place only with simultaneous participation of mechanical excitations. To simplify further analysis, the separation of unit abrasive–mechanical (I_ΔAM_), corrosion–mechanical (I_ΔCM_), and abrasive–corrosion (I_ΔAC_) interactions was abandoned. Thus, a simplified theoretical model describing wear with simultaneous abrasive, corrosive, and mechanical processes will take the form:

I_T_ = I_M_ + I_C_ + I_CA_ + I_CM_ + I_ΔACM_ (7)

Taking this into account, a series of research experiments were planned, the variants of which are summarized in Table 1.

**Figure 2:**
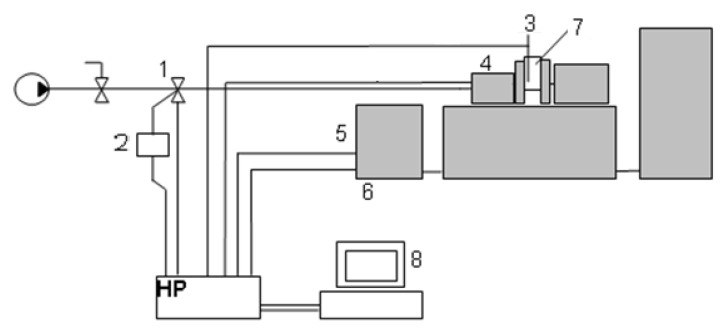
Control and measurement diagram of the UMT-2168 friction machine: 1—pneumatic valve supplying the load setting actuator; 2—pressure transducer; 3—thermocouple; 4—friction torque transducer; 5—drive system; 6—tachometer generator; 7—test chamber; 8—control panel.

The UMT-2168 test stand, a schematic diagram of which is shown in Figure 2, was used to carry out wear tests.

The tachometer generator (Join Stock Company, Iwanowo, Russia) was scaled using a revolution counter, and the thermocouple was used to measure the temperature of the near friction zone.

**Figure 3:**
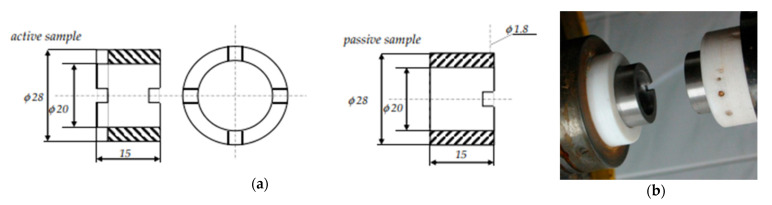
Ring-to-ring sample: (**a**) scheme with dimensions, (**b**) view of the friction node.

A ring-to-ring type node was selected for testing (Figure 3). Joints of this type are used when testing materials that replicate the operating conditions of an axial bearing, a friction sleeve joint, and for general comparative evaluation of tribotechnical properties.

**Figure 4:**
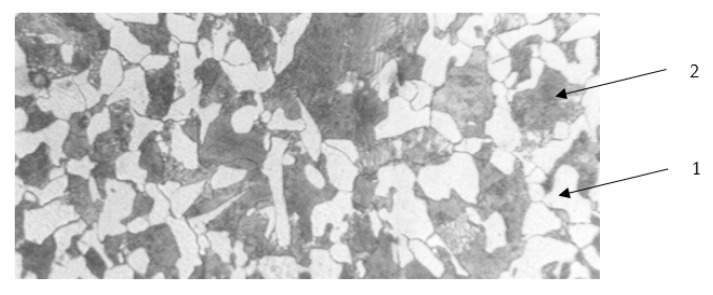
View of metallographic structures of C45 steel. 1—ferrite; 2—pearlite.

The properties of such a model joint are high-pressure uniformity and stable friction conditions at the surface in contact area. Due to the nature of the tests, structural carbon steel 45 (Table 2) was selected for the study. A view of the microstructure of the tested samples (C45 steel in non-heat-treated state) is shown in Figure 4—a mixture of ferrite and pearlite is seen. The contact surface has been prepared according to ISO class 7 (R_a_ = 0.63 µm, R_z_ = 3.2 µm).

The active sample had grooves 2 mm deep and 2 mm wide that allowed the abrasive to contact the cooperating surfaces. In addition, the passive sample had a hole for placing a thermocouple to control the temperature of near friction zone (hole at a distance of 2 mm from the cooperating surfaces). All samples were ultrasonically cleaned in extraction gasoline using an ultrasonic device, then dried and stored in a desiccator.

**Figure 5:**
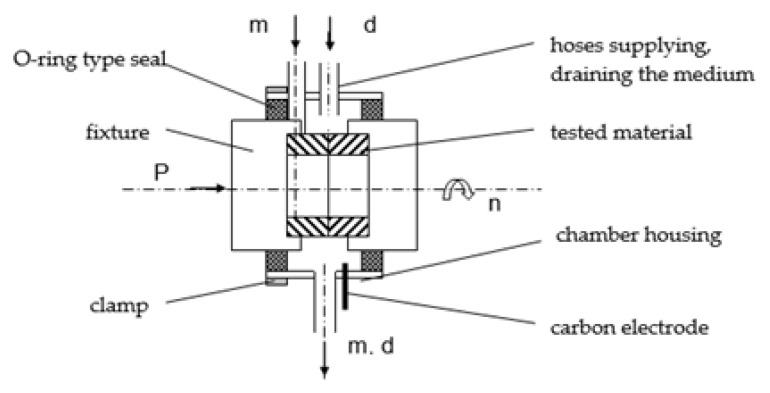
Scheme of the test chamber for corrosion test. P—pressure N; n—rotation m/s; d—abrasive of different fraction mm; m—medium.

A special test chamber was made for corrosion testing (Figure 5). The chamber for testing of abrasion–corrosion processes was made of plastic. The grips of the test material were made of polyamide, the chamber housing was made of PVC, and the medium supply and discharge hoses were made of Teflon.

Comparative tests were conducted under stabilized thermal conditions (temperature control) in distilled water with cathode protection (voltage between the test material and the carbon electrode of 1.5 V). Continuous measurement of friction torque and temperature was used to assess the quality of the process flow. A stable friction torque and temperature in a single measurement cycle qualified such an implementation for further analysis. The process duration in a single test was always 1800 s. Purified abrasive (river sand), subjected to sieve analysis (four different fractions: 0–0.05; 0.05–0.1; 0.1–0.2; 0.2–0.3), was used during the research. The chamber structure was designed to allow a continuous relatively homogeneous flow of slurry. Sulfuric acid (H_2_SO_4_) solutions were used for corrosion testing. The following solutions were used in the tests: 2%, 4%, 6%, 8%. The RADWAG PS1000/Y scale (Random, Poland) was used during the study to measure the weight loss. The weighing error was ±0.0002 g.

## 3. Results and Discussion

**Figure 6:**
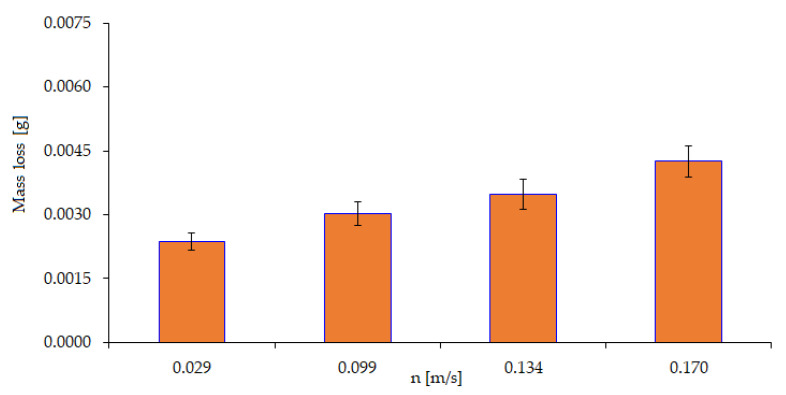
Effect of velocity on the amount of wear at a constant force P = 20 N.

The research presented in the article was designed to assess the individualized tribological and corrosion effects of the destructive processes leading to wear. The results of the tests for evaluating the mechanical wear component (implementation of variant I from Table 1) are shown in Figure 6.

**Figure 7:**
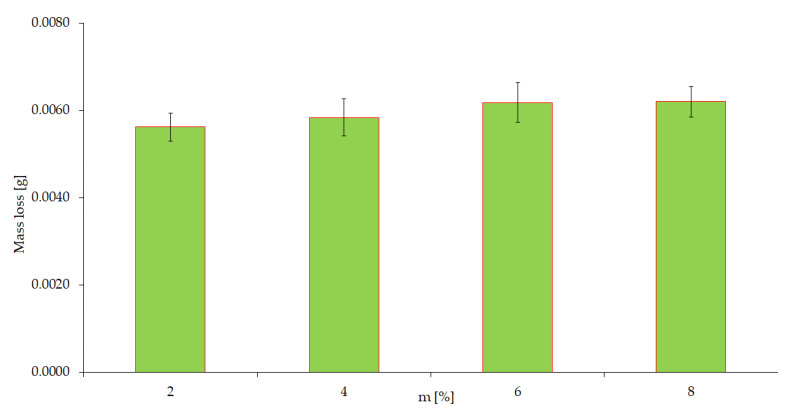
Effect of aggressive (corrosive) environment on wear results.

The results show that there is variety of wear depending on changes in test speed. An increase in speed causes a significant increase in wear. From this observation, it follows that rotational speed is a variable that can significantly affect the wear processes that occur. Experiment III (Table 1) is a methodological variant of the study involving only the corrosion factor. Figure 7 shows a graphic image of the quantitative results obtained as well as the trend of changes in corrosion wear depending on the concentration of the corrosive agent, which was sulfuric acid.

A detailed analysis of the numerical data and those visualized in the figure leads to the conclusion that corrosive factor (despite the increase in H_2_SO_4_ content) does not lead (under considered test conditions) to a significant variation in the results of corrosion wear. However, it cannot be ruled out at this stage of the research that tribocorrosive interaction processes will appear under conditions of simultaneous friction interactions.

**Figure 8:**
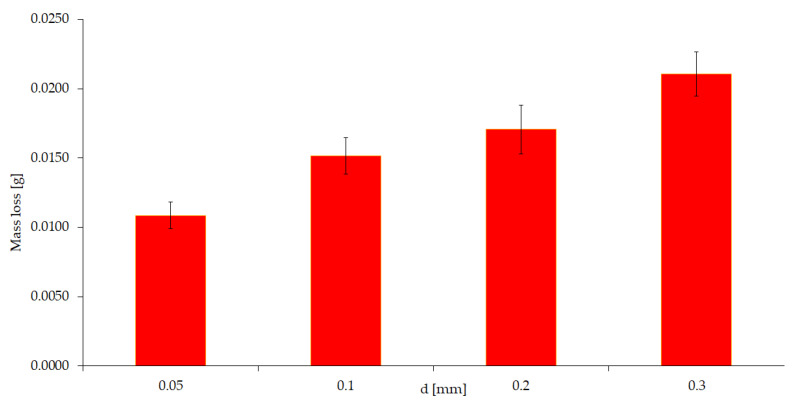
Wear value depending on abrasive grain size (without corrosion).

The experiment according to variant IV is a study with the simultaneous participation of mechanical and abrasive factors without the participation of a corrosive factor. Tests were performed for different abrasive fractions (d = 0.05; 0.1; 0.2; 0.3 mm) with constant mechanical forcing (0.1 m/s, 20 N). The results of this test are shown in Figure 8.

Based on the obtained test results (variant IV), it should be concluded that an increase in the diameter of the abrasive causes more wear. A six-fold increase in grain diameter causes twice the weight loss of the tested samples.

**Figure 9:**
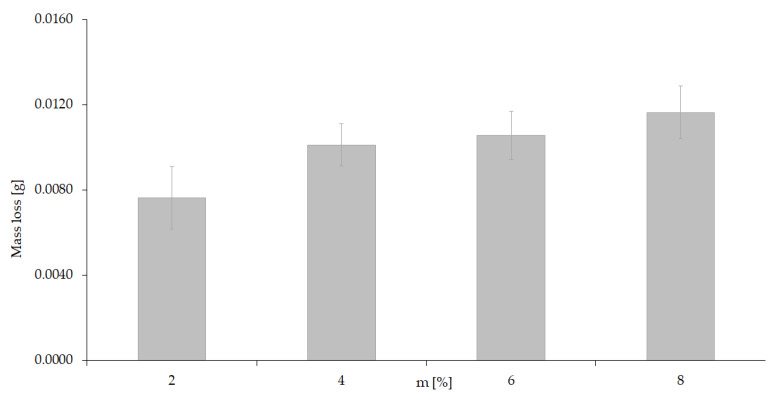
Wear value under constant mechanical forcing conditions (without abrasive) depending on the concentration of sulfuric acid.

Experiment V, according to Table 1, is a test with a mechanical (force–kinetic) factor and a corrosion factor without an abrasive factor. The results of these tests in the form of wear are shown in Figure 9.

The results of wear obtained from the tests indicate the fact of co-interaction of mechanical excitation with the aggressive factor. At the same time, the effect of the activation of corrosive interactions as the value of the aggressive factor increases can be clearly seen. Table 3 summarizes the data on the average wear of the friction node under mechanical (row 1) and corrosive (row 2) factors. The results of summing the above-mentioned wear values are shown in row 3.

The main conclusion from the analysis of the data in Table 3 can be formulated as follows: the effect of wear caused by the interaction of mechanical and corrosive excitations is not a simple sum of the wear values resulting from excitations occurring independently. This observation is the first experimental confirmation of the hypothesis of the authors of the article referring only to mechanical and corrosive interactions. In order to verify the above conclusion with regard to a more complex variant of wear, i.e., when, at the same time, the effect of final wear depends on the intensity of abrasive wear processes, additional tests were carried out. Experiment VII according to Table 1 was performed under two different assumptions:

1. With strictly defined changes in the value of the concentration of the corrosion solution, but constant values of mechanical and abrasive forcing;
2. When abrasive-induced forcing of different granularity was controlled at constant corrosive and mechanical factors.

**Figure 10:**
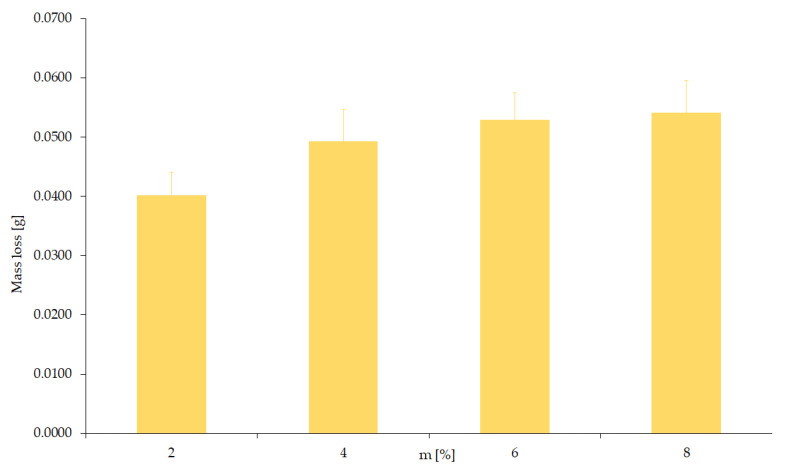
Wear value at constant values of mechanical and abrasive factors and variable concentration of corrosive agent (H_2_SO_4_).

**Figure 11:**
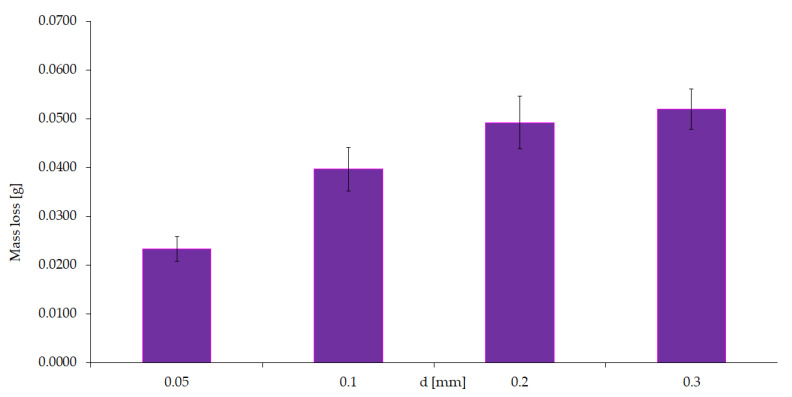
Wear volume under constant mechanical and corrosive forcing and variable abrasive characteristics.

Tests were conducted for four different concentrations of sulfuric acid (2, 4, 6, 8%) using abrasive with a size fraction in the range of 0.1–0.2 mm and mechanical forcing (0.1 m/s, 20 N)—Figure 10—and tests for four different abrasive fractions (0.05; 0.1; 0.2; 0.3 mm) at a constant concentration of 4% H_2_SO_4_ and mechanical forcing (0.1 m/s, 20 N)—Figure 11.

**Figure 12:**
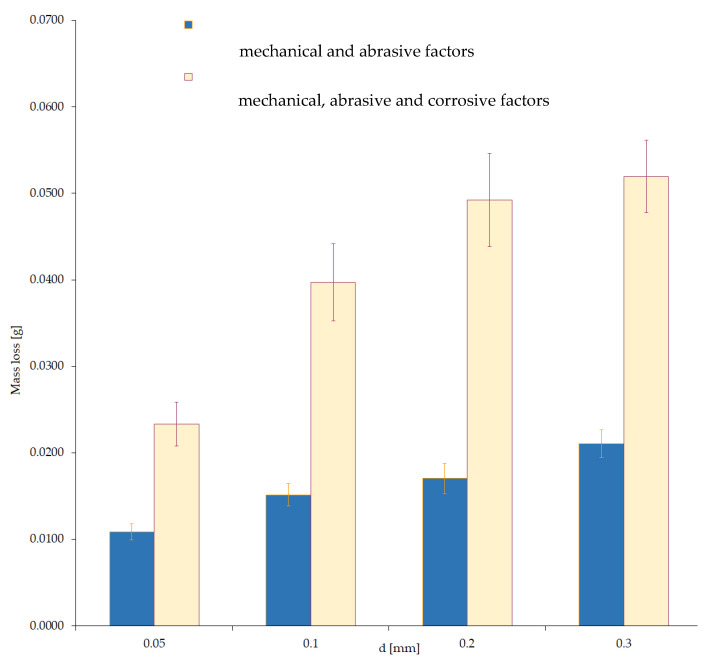
Wear value depending on the variable grain size of the abrasive, under constant mechanical and mechanical–corrosion interactions.

The presented tests result made it possible to illustrate the changes in the effects of total wear, which were obtained with the presence of mechanical and abrasive, as well as mechanical–abrasive–corrosive excitations (Figure 12). These results show the significant contribution of the wear component, which depends on the amount of the corrosive factor and the effects of the interaction of all sources of tribological forcing.

**Figure 13:**
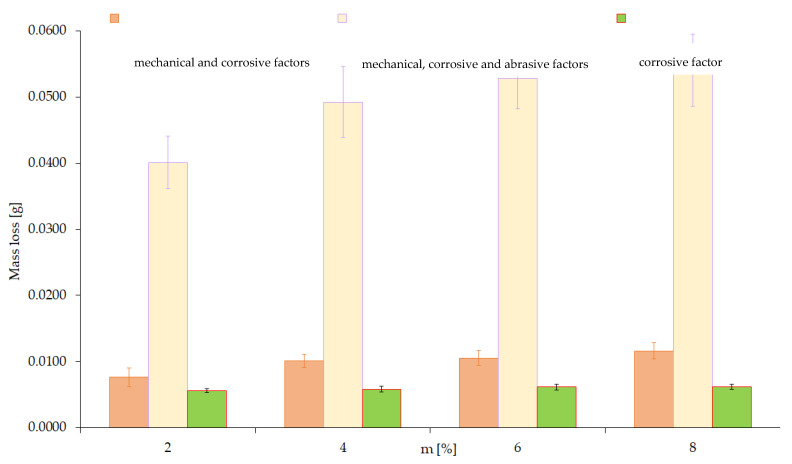
Comparison of the effects of the influence of the corrosive factor with the effects of simultaneous mechanical and corrosive factors and total wear caused by mechanical, abrasive, and corrosive interactions.

A comparison of the obtained results from wear tests with the interaction of a corrosive agent, mechanical–corrosive agents and mechanical–corrosive–abrasive interactions is shown in Figure 13. From the data in this figure, it is clear that the interaction effect caused by abrasive interactions is significant. The presented results clearly show that total wear is not a simple superposition of the individualized effects of friction and corrosive processes. Thus, the conclusion that the wear effect caused by the interaction of mechanical and corrosive excitations is not a simple sum of wear values resulting from excitations occurring independently was confirmed experimentally.

Based on the results, it should be concluded that the size of the abrasive particle has a significant impact on the mass loss (wear), which confirms the conclusions of the paper [29]. Larger abrasive particles result in the removal of a larger volume of material during destructive impact on the steel surface. The wear, depending on the abrasive particle, is at a level of about 0.01 g to 0.02 g, which is higher than the wear in the work [27], which was about 0.006 g. Wieczorek [30] tested the influence of abrasive materials on the wear of hard-wearing steels. The resistance of wear-resistant steels was found to be about four times higher than that of structural steel S355J2 for quartz abrasive.

**Figure 14:**
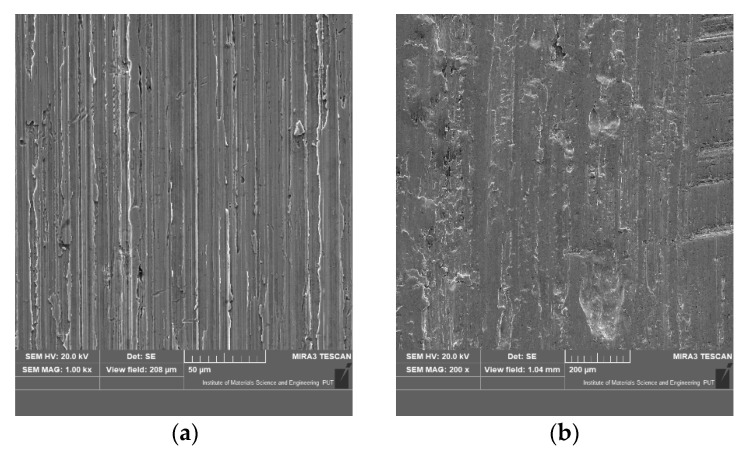
SEM morphology of worn surfaces of C45 steel for abrasive and mechanical wear (**a**,**b**).

The authors of the paper [31] studied the influence of different degradation mechanisms occurring during corrosion–abrasion tests. For neutral test conditions, sliding abrasion significantly affected the wear of martensitic stainless steel. Nevertheless, lowering the pH changed the wear-inducing factor. The dominant role was taken by corrosion and the synergistic effect between corrosion and abrasion wear. Similar results were obtained by the authors of this article, where the addition of a corrosive factor significantly affected the result of wear (mass loss) compared to the situation where only the mechanical factor and abrasive wear interacted on the tested surface of the samples. The addition of a corrosive agent causes twice as much wear (mass loss) compared to the effect of only mechanical agents and abrasive. The surface of the sample after wear process was presented in Figure 14.

Example views of the surface after exposure to destructive agents show characteristic wear marks due to the action of abrasive particles. There was micro-cutting and micro-scratching of the surface due to the action of the abrasive particles and the pressing force on the surfaces of the moving parts. During the study of wear processes, the friction torque was recorded for various destructive factors. A summary of the results of the average friction torque is shown in Table 4, Table 5, Table 6, Table 7 and Table 8.

The compiled results of the average friction torques during the implementation of the tests are in the range of about 0.1 to 2.9 Nm. These values are consistent with other results presented in literature [32]. It is clear that the friction torque depends on the size of the abrasive—the larger the abrasive particles, the more the friction torque increases. In addition, the increase in frictional torque is caused by a higher concentration of the corrosive agent.

## 4. Conclusions

Taking into account the obtained experimental results of wear for different processes, the following conclusions can be made:

- The sum component of mechanical–abrasive–abrasive interactions is, in most cases, in the range of 40–50% of the total wear;
- In total wear, the component resulting only from mechanical interactions (friction), is at the level of a few to several percent;
- At a slightly higher level of several percent, the values of the corrosive component are formed;
- The values of wear resulting from mechanical and corrosive interactions and corrosion are at the level of a few percent. In one variant of the study, the effect of individual factors of a synergistic nature was not recorded;
- The share of the component resulting from simultaneous mechanical and abrasive interactions is, on average, about one-third of the total wear.

Taking into account the last two conclusions, it is possible to formulate the observation that abrasive processes and the interaction processes caused by them play a dominant role in abrasive–corrosion wear. Thus, that the veracity of the formulation considered in destructive interactions, abrasive processes, and their interacting abrasive–mechanical, abrasive–corrosion, and abrasive–corrosion–mechanical interactions plays a dominant role is confirmed.

Presented in this article are research results of wear of machine elements made of C45 steel occurring due to the simultaneous action of mechanical–abrasive–corrosive factors. These processes are characteristic of machine nodes and equipment used in technological processes carried out in many branches of the food industry. On the basis of the performed research, it can be concluded that the total wear that takes place in tribological nodes due to the simultaneous occurrence of destructive processes: friction (mechanical), corrosive, and abrasive is not a simple superposition of their individualized effects occurring under conditions of their independent factors. It was observed that the effects of the interaction of these three factors have a synergistic character—an increase in the total wear of the tested samples. The results of the study confirm the hypothesis that in the complex conditions of mechanical, corrosive, and abrasive forcing, there are interactions, the intensity of which depends mainly on the nature of the intensity of abrasive processes.

## Figures and Tables

**Table 1:** Variants of implementation of mechanical–abrasive–corrosive tests.

Experiment	Extortion-Factor			Model
	Mechanical	Abrasive	Corrosive	
	P = 20 N n = 0.1 m/s	d = 0.2 mm	m = 4% H_2_SO_4_	
I	X	-	-	I_I_ = I_M_
II	-	X	-	-
III	-	-	X	I_III_ = I_C_
IV	X	X	-	I_IV_ = I_M_ + I_A_ + I_Δ__AM_
V	X	-	X	I_V_ = I_M_+ I_C_ + I_Δ__MC_
VI	-	X	X	-
VII	X	X	X	I_VII_ = I_C_^E^ = I_M_ + I_C_ + I_A_ + I_Δ_

X—the factor present in an experiment. -—the factor absent in an experiment.

**Table 2:** Characteristics of structural carbon steel 45.

Chemical Composition					
C	Mn	Si	P_max_	S_max_	Fe
%	%	%	%	%	%
0.42–0.50	0.50–0.80	0.17–0.37	0.040	0.040	rest
**Mechanical properties**					
UTS	YS	Elongation	Hardness		
MPa	MPa	%_min_	HV10		
610	275	16	240		

**Table 3:** Summary of the results of wear tests with the impact of mechanical factor alone, corrosion alone, and mechanical–corrosion (without the impact of abrasive).

Variant		2%	4%	6%	8%
		I [g]	I [g]	I [g]	I [g]
I	I_M_	0.0030	0.0030	0.0030	0.0030
II	I_C_	0.0056	0.0058	0.0062	0.0062
	Σ(I_M_ + I_C_)	0.0086	0.0088	0.0092	0.0092
V	I_Δ__CM_	0.0076	0.0101	0.0106	0.0116

**Table 4:** Average friction torque values for mechanical wear without the influence of abrasive and corrosive agents.

Diameter of Abrasive Particles	m	Velocity	Force	Average Friction Torque (AFT)	ΔAFT
mm	%	rpm	N	Nm	Nm
0	0	23	20	0.103	0.007
0	0	80	20	0.116	0.004
0	0	108	20	0.148	0.014
0	0	133	20	0.205	0.011

**Table 5:** Average friction torque values for test results with constant mechanical factors and with variable abrasive action (without corrosive action).

Diameter of Abrasive Particles	m	Vezlocity	Force	Average Friction Torque (AFT)	ΔAFT
mm	%	rpm	N	Nm	Nm
0.05	0	80	20	1.234	0.077
0.1	0	79	20	1.789	0.166
0.2	0	79	20	2.414	0.212
0.3	0	78	20	3.232	0.208

**Table 6:** Average friction torque values for test results under constant mechanical forcing with variable corrosive action (without abrasive action).

Diameter of. Abrasive Particles	m	Velocity	Force	Average Friction Torque (AFT)	ΔAFT
mm	%	rpm	N	Nm	Nm
0	2	80	20	1.054	0.058
0	4	79	20	1.133	0.108
0	6	80	20	1.17	0.068
0	8	80	20	1.286	0.064

**Table 7:** Average friction torque values for tests results with constant mechanical factors with variable corrosive action and with 0.2 mm abrasive.

Diameter of Abrasive Particles	m	Velocity	Force	Average Friction Torque (AFT)	ΔAFT
mm	%	rpm	N	Nm	Nm
0.2	2	79	21	1.72	0.147
0.2	4	79	20	2.02	0.133
0.2	6	80	20	2.184	0.074
0.2	8	79	20	2.291	0.128

**Table 8:** Average friction torque values for test results for constant mechanical interactions with variable abrasive action in 4% sulfuric acid.

Diameter of Abrasive Particles	m	Velocity	Force	Average Friction Torque (AFT)	ΔAFT
mm	%	rpm	N	Nm	Nm
0.05	4	79	20	0.98	0.044
0.1	4	79	21	1.242	0.098
0.2	4	79	21	2.02	0.133
0.3	4	79	21	2.92	0.133

## Data Availability

Not applicable.
